# Inhibition of miR-199a-5p rejuvenates aged mesenchymal stem cells derived from patients with idiopathic pulmonary fibrosis and improves their therapeutic efficacy in experimental pulmonary fibrosis

**DOI:** 10.1186/s13287-021-02215-x

**Published:** 2021-02-25

**Authors:** Linli Shi, Qian Han, Yimei Hong, Weifeng Li, Gencheng Gong, Jiangyu Cui, Mengmeng Mao, Xiaoting Liang, Bei Hu, Xin Li, Qun Luo, Yuelin Zhang

**Affiliations:** 1 The Second School of Clinical Medicine, Southern Medical University, Guangzhou, 510080 Guangdong China; 2 Department of Emergency Medicine, Guangdong Provincial People’s Hospital, Guangdong Academy of Medical Sciences, Guangzhou, Guangdong China; 3 Department of Respiratory Medicine, The First Affiliated Hospital of Guangzhou Medical University, Guangzhou Institute of Respiratory Health, State Key Laboratory of Respiratory Disease, Guangzhou, Guangdong China; 4 Institute of Regenerative Medicine, Shanghai East Hospital, Tongji University School of Medicine, Shanghai, China

**Keywords:** Mesenchymal stem cells, miR-199a-5p, Rejuvenation, Senescence, Idiopathic pulmonary fibrosis

## Abstract

**Background:**

Idiopathic pulmonary fibrosis (IPF) is an age-related disease with no cure. Mesenchymal stem cell (MSC)-based therapy has emerged as a novel strategy for IPF treatment. Nevertheless, MSCs derived from patients with IPF (IPF-MSCs) become senescent, thereby reducing their beneficial effects in IPF. MicroRNAs (miRNAs) mediate the senescence of MSCs, but the underlying mechanisms are not fully understood. We investigated the mechanisms by which miR-199a-5p regulates IPF-MSC senescence and whether its inhibition could rejuvenate IPF-MSCs and enhance their therapeutic efficacy.

**Methods:**

Control-MSCs and IPF-MSCs were isolated from the adipose tissue of age-matched healthy and IPF donors, respectively. Cell senescence was examined by senescence-associated β-galactosidase (SA-β-gal) staining. The level of miR-199a-5p was measured by RT-PCR. Autophagy was determined using a transmission electron microscope (TEM). The therapeutic efficacy of anti-miR-199a-5p-IPF-MSCs was assessed using a mouse model of bleomycin-induced lung fibrosis.

**Results:**

Despite similar surface makers, IPF-MSCs exhibited increased cellular senescence and decreased proliferative capacity compared with control-MSCs. The expression of miR-199a-5p was significantly enhanced in the serum of IPF patients and IPF-MSCs compared with that of healthy donors and control-MSCs. The upregulation of miR-199a-5p induced senescence of control-MSCs, whereas the downregulation rescued IPF-MSC senescence. Mechanistically, miR-155-5p suppressed autophagy of MSCs via the AMPK signaling pathway by downregulating the expression of Sirtuin 1(Sirt1), resulting in cellular senescence. Accordingly, miR-155-5p inhibition promoted autophagy and ameliorated IPF-MSC senescence by activating the Sirt1/AMPK signaling pathway. Compared with IPF-MSCs, the transplantation of anti-miR-199a-5p-IPF-MSCs increased the ability to prevent progression of pulmonary fibrosis in bleomycin-treated mice.

**Conclusions:**

Our study shows that miR-199a-5p regulates MSC senescence in patients with IPF by regulating the Sirt1/AMPK signaling pathway and miR-199a-5p is a novel target to rejuvenate IPF-MSCs and enhance their beneficial effects.

**Supplementary Information:**

The online version contains supplementary material available at 10.1186/s13287-021-02215-x.

## Introduction

Idiopathic pulmonary fibrosis (IPF) is characterized by chronic and progressive fibrosing interstitial pneumonia. It is a life-threatening disease that mainly affects middle-aged and older adults [1, 2]. The annual incidence is estimated to be 4.6 to 16.3 cases per 100,000 worldwide, and median survival after diagnosis is approximately 3 to 5 years [3]. Although pharmacological therapies such as pirfenidone and nintedanib may limit IPF, the only cure is lung transplantation [4]. This is nonetheless limited by its high cost, a shortage of available donor organs, and immunorejection post transplantation. A novel strategy to treat IPF is urgently needed.

Over the past decades, a growing body of evidence from animal studies and clinical trials has demonstrated that mesenchymal stem cell (MSC)-based therapy is a potential novel approach for IPF [5–7]. MSCs can exert multiple protective effects in the lung with pulmonary fibrosis that include decreasing inflammation, reducing collagen deposition, differentiating into local cell types, and activating resident stem cells [8, 9]. Nonetheless, the function of MSCs declines with age with a reduction in their capacity for tissue repair [10–12]. Compared with age-matched control-MSCs, those isolated from IPF patients (IPF-MSCs) exhibit more senescence, manifested by DNA damage, dysfunctional mitochondria, and decreased function, leading to a reduced therapeutic effect for IPF in mice [13]. Nevertheless, the potential mechanisms underlying IPF-MSC senescence are unclear.

MicroRNAs (miRNAs), a class of ~ 21–23 nucleotide long noncoding RNAs, are critical repressors of gene expression, achieved by binding to the 3′-untranslated region (UTR) of target mRNAs. Previous studies have shown that miRNAs are involved in mediating MSC senescence via regulation of multiple signaling pathways [14, 15]. Our previous study showed that miR-155-5p inhibits mitochondrial fission in MSCs via the Cab39/AMPK signaling pathway, resulting in cellular senescence. Inhibition of miR-155-5p has been shown to rejuvenate aged-MSCs and enhance their cardioprotective effects on myocardial infarction in mice [16]. It has been reported that the level of miR-199a-5p is significantly increased in patients with IPF and in mice with bleomycin-induced lung fibrosis [17]. This altered miR-199a-5p expression prompted the search for a potential relationship between miR-199a-5p and IPF-MSC senescence. Nonetheless, whether and how miR-199a-5p regulates the cellular senescence of MSCs in IPF has not been investigated.

Autophagy plays a critical role in maintaining cellular homeostasis via degradation of harmful cytoplasmic components by autolysosomes. Recent studies have shown that failure of autophagy is closely associated with cellular senescence of stem cells, including MSCs [18, 19]. MSCs isolated from aged-donors display deficient autophagy. Macrophage migration inhibitory factor rejuvenates these aged-MSCs by activating autophagy [20]. Whether miR-199a-5p mediates IPF-MSC senescence by regulating autophagy and the potential underlying mechanisms nonetheless remain unclear. In the current study, we investigated the role of miR-199a-5p in the regulation of IPF-MSC senescence and explored the underlying molecular mechanisms. We also investigated whether inhibition of miR-199a-5p could rejuvenate IPF-MSCs and improve their therapeutic effects in a mouse model of IPF.

## Methods

### Cell culture

Adipose-derived MSCs were isolated from healthy donors (59.5 ± 5.4 years; *n* = 7) and IPF patients (60.5 ± 3.02 years; *n* = 6). Written informed consent was obtained from all donors. The procedure was approved by the research ethics board of The First Affiliated Hospital of Guangzhou Medical University. The demographic information of study subjects is summarized in Table 1. The adipose tissue (1–5 g) was washed three times with PBS, cut into small pieces, and digested with enzyme and subsequently plated on 10 cm culture dishes. After 48 h, non-adherent cells were washed off and the remainder cultured at 37 °C in DMEM/high glucose (1196508, Gibco, USA) supplemented with 10% FBS (16000, Life Technologies, USA), 5 ng/ml EGF (AF-100-15, PeProTech, USA), and 5 ng/ml FGF2 (100-18B, PeProTech, USA) in a humidified atmosphere with 5% CO_2_. The medium was changed every 48 h. All MSCs used in the current study were at passage 3–4. Both control-MSCs and IPF-MSCs were passaged at 3-day intervals and the same cell number (100,000 cells per 6-cm dish) plated. Population doubling was determined at each passage.

**Table 1:** Demographic characteristics of the study subjects

Total subjects	IPF	Control	***p*** value
	**6**	**7**	–
Males/females (*n*)	6/0	6/1	–
Age (year), mean ± SEM	60.5 ± 3.02	59.5 ± 5.4	0.775
Disease duration (year), mean ± SEM	1.9 ± 0.8	–	–
FVC (L), mean ± SEM	2.4 ± 0.7	3.9 ± 0.3	< 0.001
FVC%, mean ± SEM	65.9 ± 13	92.7 ± 5.2	<0.001
DLCO%, mean ± SEM	47.4 ± 7.4	97.1 ± 5.4	0.003
Location of the fat biopsy	Subcutaneous	Subcutaneous	–

*IPF* idiopathic pulmonary fibrosis, *FVC* forced vital capacity, *DLCO* carbon monoxide diffusing capacity

### Characterization of MSCs

The surface markers of control-MSCs and IPF-MSCs were evaluated by flow cytometry. The following antibodies were used: anti-CD34 (343607, BioLegend, San Diego, CA), anti-CD45 (304011, BioLegend, San Diego, CA), anti-CD73 (344003, BioLegend, San Diego, CA), anti-CD90 (328107, BioLegend, San Diego, CA), and anti-CD105 (323205, BioLegend, San Diego, CA). The differentiation capacity of control-MSCs and IPF-MSCs into adipocytes, osteocytes, and chondrocytes was evaluated as described previously [21].

### Western blotting

The proteins of MSCs were extracted using a total protein extraction kit (BB-3101, Bestbio, Shanghai, China), and their concentration determined using a bicinchoninic acid (BCA) assay kit (231227, Thermo, MA, USA). A total of 25 μg protein from each sample was loaded in each lane and separated by sodium dodecyl sulfate-polyacrylamide gel electrophoresis. Subsequently, the proteins were transferred onto PVDF membranes. After blocking with 5% fat-free milk in TBST, the membranes were incubated overnight at 4 °C with the following primary antibodies: anti-p16 (1:1000, ab51243, Abcam, Cambridge, UK), anti-p21 (1:1000, ab109199, Abcam, Cambridge, UK), anti-Beclin (1:1000, 3738, CST, MA, USA), anti-LC3I/II (1:1000, 4108, CST, MA, USA), anti-p62 (1:1000, 5114, CST, MA, USA), anti-AMPK (1:1000, 5832, CST, MA, USA), anti-p-AMPK (1:1000, 4184, CST, MA, USA), anti-Sirt1 (1:1000, ab110304, Abcam, Cambridge, UK), anti-α-SMA (1:100, ab5694, Abcam, Cambridge, UK), anti-collagen I (1:100, ab34710, Abcam, Cambridge, UK), and GAPDH (1:1000, 2118, CST, MA, USA). The membranes were then washed three times with TBST and treated with the relevant secondary antibody (1:1000, CST, MA, USA) for at least 1 h at room temperature and exposed to radiography film in a dark room.

### SA-β-gal assay

The cellular senescence of MSCs was determined using a SA-β-gal assay kit (C0602, Beyotime, Shanghai, China). The MSCs at passages 3 ~ 4 were used for SA-β-gal assay in the current study. Briefly, the same number of control-MSCs and IPF-MSCs with different treatments was cultured in 6-well plates. Subsequently, MSCs were fixed for 15 min after washing with PBS and then incubated with an SA-β-gal staining reagent overnight at 37 °C without CO_2_. Finally, the samples were washed and three randomly selected fields were imaged with a microscope. The percentage of senescent MSCs was determined by the ratio of blue (positive) MSCs to all MSCs obtained from five different view fields of each sample.

### Bromodeoxyuridine (Brdu) incorporation assay

The proliferation capacity of MSCs was determined sing a BrdU incorporation kit according to the manufacturer’s instructions (1164722900, Roche, Basel, Switzerland). Briefly, 3 × 10^4^ MSCs were plated in 96-well microplates and cultured with 10 μM BrdU labeling solution for 24 h at 37 °C. After removal of labeling medium, MSCs were incubated with 200 μl FixDenat solution for 30 min and then treated with anti-BrdU-POD working solution for 90 min. Subsequently, MSCs were washed with PBS three times and incubated with 100 μl substrate solution for 5 min. Absorbance at 450 nm was then determined.

### Immunofluorescence staining

After fixing with 4% paraformaldehyde (P0099, Beyotime, Shanghai, China) for 30 min, MSCs were washed with PBS and permeated with 0.1% Trion X-100, then incubated overnight at 4 °C with anti-Ki67 antibody (1:100, ab15580, Abcam, Cambridge, UK) and *γ*H2AX antibody (1: 100, ab81299, Abcam, Cambridge, UK). Subsequently, MSCs were incubated in the dark with the fluorescent-labeled secondary antibodies (1:1000) for 1 h after being washed three times with PBS (10 min each) and finally mounted with DAPI to stain the nucleus and imaged with a fluorescence microscope. The percentage of Ki67-positive cells was calculated as the ratio of Ki67-positive MSCs to all DAPI-positive cells.

### Transfection of miR-199a-5p mimic and inhibitor

The miR control, miR-199a-5p mimics, and miR-199a-5p inhibitors were purchased from GenePharma (Shanghai, China). MSCs were transfected with miR control, miR-199a-5p mimic, or miR-199a-5p inhibitor (50 nM) using a Lipofectamine RNAiMAX Reagent Kit (2145966, Invitrogen, California, USA) according to the manufacturer’s instructions. The MSCs were cultured at 37 °C in a 5% CO_2_ incubator following transfection and harvested 48 h later for further experiments. Transfection was performed at least three times.

### qRT-PCR

Total RNA, including miRNAs, was isolated from MSCs, serum or lung tissue with TRIzol reagent followed by RNase-free DNase I (2270A, Takara, Tokyo, Japan) treatment. Reverse transcription was performed with a PrimeScript RT Reagent Kit (RR037A, Takara, Tokyo, Japan). The assays of Taqman miRNA were used to quantify the expression level of miR-199a-5p (002623, Applied Biosystems, CA, USA). U6 was the reference gene for miRNA expression analysis. The expression of miR-199a-5p was normalized to U6 expression by the 2-ΔΔCt method. Measurement of mRNA levels was made using SYBR Green Master Mix (Q111-02, Vazyme, Nanjing, China) after reverse transcription of 1 μg RNA into the first-strand cDNA. The expression of each mRNA in different groups was normalized to GAPDH and calculated using the 2-ΔΔCt method. The primer sequences used to amplify the mouse RNA were as follows: collagen I forward primer, 5′-ATCAGCTGGAGTTTCCGTGC-3′ and collagen I reverse primer, 5′-CTGTTCCAGGCAATCCACGA-3′; α-SMA forward primer, 5′-GGCATCCACGAAACCACCTA-3′ and α-SMA reverse primer, 5′-TTCCTGACCACTAGAGGGGG-3′; IL-1β forward primer, 5′-TGCCACCTTTTGACAGTGATG-3′ and IL-1β reverse primer, 5′-AAGGTCCACGGGAAAGACAC-3′; IL-6 forward primer, 5′-CAACGATGATGCACTTGCAGA-3′ and IL-6 reverse primer, 5′-TGTGACTCCAGCTTATCTCTTGG-3′; IL-8 forward primer, 5′-CTAGGCATCTTCGTCCGTCC-3′ and IL-8 reverse primer, 5′-CAGAAGCTTCATTGCCGGTG-3′. The experiments were repeated at least three times. Human Alu-sx repeat sequences were detected by genomic PCR to determine MSC survival in lung tissue from the different groups. The primer of human Alu-sx was F:5′-GGCGCGGTGGCTCACG-3′, R:5′-TTTTTTGAGACGGAGTCTCGCTC-3. The product was evaluated by electrophoresis in 1.5% agarose gel supplemented with ethidium bromide.

### Transmission electron microscope (TEM)

Autophagosomes of MSCs were examined using a TEM. In brief, after washing with PBS, cells were fixed with 2.5% glutaraldehyde in phosphate buffer for 4 h and then post-fixed for 2 h with 1% OsO_4_ in the same buffer. Next, cells were dehydrated in a graded ethanol series (30, 50, 70, 80, 90, 95, and 100%). Subsequently, cells were infiltrated with 1:1 acetone: Spurr resin (02690-AB, SPI-Chem, PA, USA) for 1 h at room temperature, 1:3 acetone: Spurr resin for 3 h, and then absolute Spurr resin overnight. Images were captured for further analysis using a TEM (H-7650, Hitachi,).

### siRNA and lentiviral transduction

MSCs were transfected with Sirt1-siRNA (RXO13987, TranSheepBio, Shanghai, China) or control siRNA (RXO13004, TranSheepBio, Shanghai, China) using a Lipofectamine RNAiMAX Reagent Kit (2145966, Invitrogen, California, USA) according to the manufacturer’s protocol. The lentiviral plasmid constructs for overexpression of Sirt1 in control-MSCs were purchased from TranSheepBio (Shanghai, China; Fig. S1A). The lentiviral plasmid constructs for inhibition of miR-199a-5p in IPF-MSCs were purchased from GenePharma (Shanghai, China, Fig. S1B). The lentivirus was packaged as per the manufacturer’s protocol. MSCs at a confluence of 70–80% were infected by lentivirus at a multiplicity of infection of 10 with polybrene (8 μg/ml). The transfection efficiency was determined after 72 h by Western blotting and PCR.

### IPF model establishment and transplantation of MSCs

All animal experiments were performed at the Laboratory Animal Center of Guangzhou Yongnuo and approved by the Animal Ethical and Welfare Committee (AEWC) of Yongnuo Medical Laboratory Animal Center (No. IACUC-G16021). The IPF model was induced in C57BL/6J male mice (8–10 weeks old, weighing 23–28 g) by direct injection into the trachea of 2 U/kg bleomycin hydrochloride (390320, Hanhui Pharmaceutical, Hangzhou, China) solution using a 0.9-mm needle after anesthesia with 1% sodium pentobarbital. Mice were randomly divided into five groups: control group, bleomycin group, bleomycin + control-MSC group, bleomycin + IPF-MSCs group, and bleomycin + anti-miR-199a-5p IPF-MSCs group (*n* = 6 in each group). Twenty-four hours following bleomycin administration, 5 × 10^5^ human adipose-MSCs from different groups suspended in 100ul PBS were intravenously injected into the mice via tail vein. At 14 days post-transplantation, mice were deeply anesthetized with 1% sodium pentobarbital and intrapulmonary blood was replaced by saline through cardiac perfusion. Subsequently, mice succumbed due to the excessive blood loss and the lungs were harvested. The histopathology and fibrosis deposition of the lung tissue from different groups were analyzed by HE staining and Masson’s trichrome staining, respectively. Fibrotic areas occupied by collagen (blue) were quantified using Image-Pro Plus software (Version X; Adobe, San Jose, CA). The percentage of fibrosis was calculated for each mouse as the ratio of the fibrotic area to the total area × 100%. At least 5 lung parenchyma were counted in each slide.

### Statistical analysis

Statistical analyses were performed using Prism 5.0 (GraphPad Software), and results expressed as the mean ± SEM. Differences between two groups were analyzed by unpaired Student’s *t* test and multiple groups by one-way ANOVA followed by Bonferroni test. A value of *p* < 0.05 was considered statistically significant.

## Results

### Characterization of control-MSCs and IPF-MSCs

**Fig. 1:**
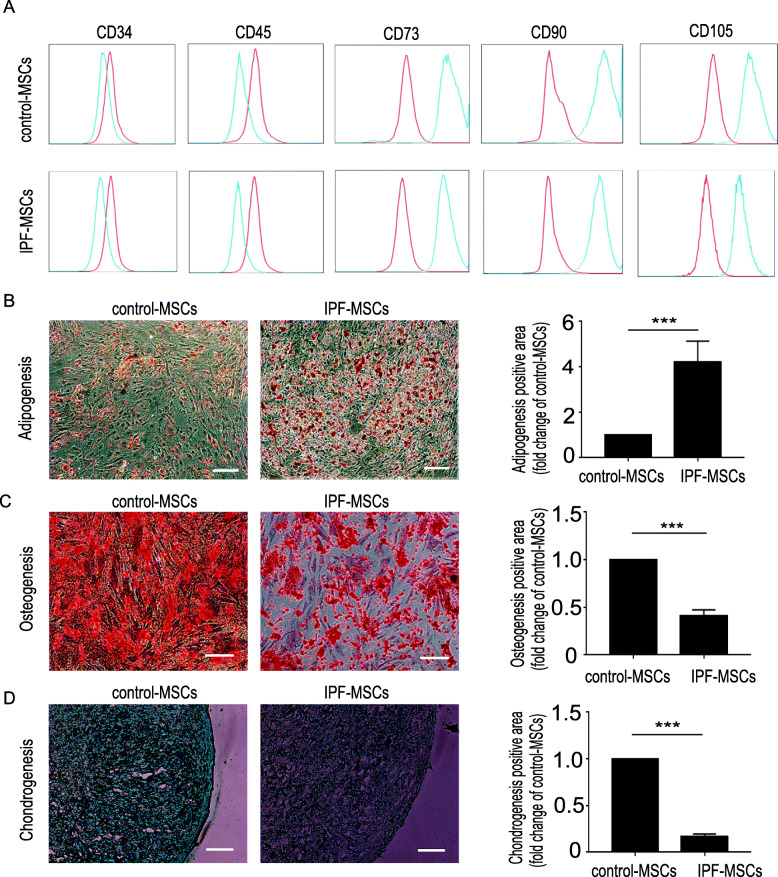
Characterization of control-MSCs and IPF-MSCs. **a** Surface markers of control-MSCs and IPF-MSCs were evaluated by flow cytometry. Both control-MSCs and IPF-MSCs expressed CD73, CD90, and CD105 but not CD34 or CD45. **b** Adipogenic differentiation evaluated by Oil Red staining and quantification of adipogenic efficiency in control-MSCs and IPF-MSCs. **c** Osteogenic differentiation determined by Alizarin red staining and quantification of osteogenic efficiency in control-MSCs and IPF-MSCs. **d** Chondrogenic differentiation determined by Alcian blue staining and quantification of chondrogenic efficiency in control-MSCs and IPF-MSCs. All data were obtained from at least three independent experiments and each error bar represents the mean ± SEM. Scale bar = 200 μm. ***p < 0 .001

We first examined the surface antigens of control-MSCs and IPF-MSCs using flow cytometry. Both control-MSCs and IPF-MSCs had similar surface markers. They were CD73, CD90, and CD105-positive but CD34 and CD45-negative (Fig. 1a). Next, we examined the capacity of control-MSCs and IPF-MSCs to differentiate into adipocytes, osteocytes and chondrocytes. Both types of MSCs could differentiate into adipocytes, as evidenced by Oil Red O staining. Strikingly, IPF-MSCs showed a distinctly higher adipogenic differentiation capacity than control-MSCs (Fig. 1b) but a significantly lower osteogenic and chondrogenic differentiation capacity, as evidenced by Alizarin Red and Alcian blue staining respectively (Fig. 1c, d). These results suggest that the differentiation capacity of IPF-MSCs was altered.

### IPF-MSCs are more senescent than control-MSCs

**Fig. 2:**
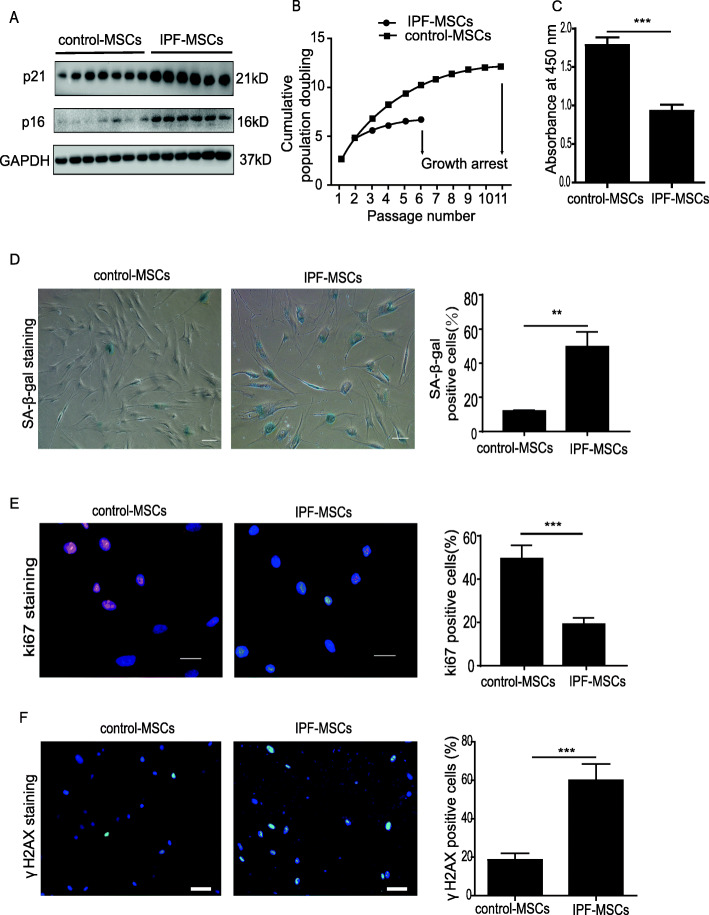
IPF-MSCs displayed increased cellular senescence. **a** Western blotting analysis of the expression of p21 and p16 in control-MSCs and IPF-MSCs. **b** Cell growth curves demonstrate the lower proliferative ability of IPF-MSCs compared with control-MSCs. **c** BrdU assay shows a decreased absorbance at 450 nm of IPF-MSCs compared with control-MSCs. **d** Representative images of SA-β-gal staining and quantitative analysis of SA-β-gal-positive cells in control-MSCs and IPF-MSCs. Scale bar = 200 μm. **e** Immunostaining of the proliferation marker Ki67 and quantitative analysis of Ki67-positive cells in control-MSCs and IPF-MSCs. Scale bar = 100 μm. **f** Immunostaining of the DNA damage marker γH2AX and quantitative analysis of γH2AX-positive cells in control-MSCs and IPF-MSCs. Scale bar = 200 μm. All data were obtained from at least three independent experiments and each error bar represents the mean ± SEM. **p < 0.01; ***p < 0.001

Since IPF-MSCs displayed an altered differentiation potential, we next examined whether they were senescent. The protein level of cellular senescence markers including p21 and p16 in MSCs derived from six IPF patients was significantly higher than that of seven healthy individuals (Fig. 2a). Next, we evaluated the cell growth rate of control-MSCs and IPF-MSCs via serial passaging. As shown in Fig. 2b, the proliferation of IPF-MSCs was slow and arrested at passage 6, whereas control-MSCs continued growing until passage 11, indicating that the proliferation of IPF-MSCs was decreased (Fig. 2b). BrdU assay also showed that compared with control-MSCs, the absorbance at 450 nm of IPF-MSCs was significantly reduced, indicating a decreased proliferative rate (Fig. 2c). Furthermore, compared with control-MSCs, the percentage of SA-β-gal-positive cells was dramatically increased in IPF-MSCs (Fig. 2d). We then assessed the proliferative rate using Ki67 staining and found a lower proliferative ability of IPF-MSCs compared with control-MSCs (Fig. 2e). We also examined DNA damage in control-MSCs and IPF-MSCs using γH2AX staining. The percentage of γH2AX-positive cells was greatly increased in IPF-MSCs compared with control-MSCs (Fig. 2f). Collectively, these data indicate that IPF-MSCs showed more cellular senescence.

### miR-199a-5p mediates cellular senescence of IPF-MSCs

**Fig. 3:**
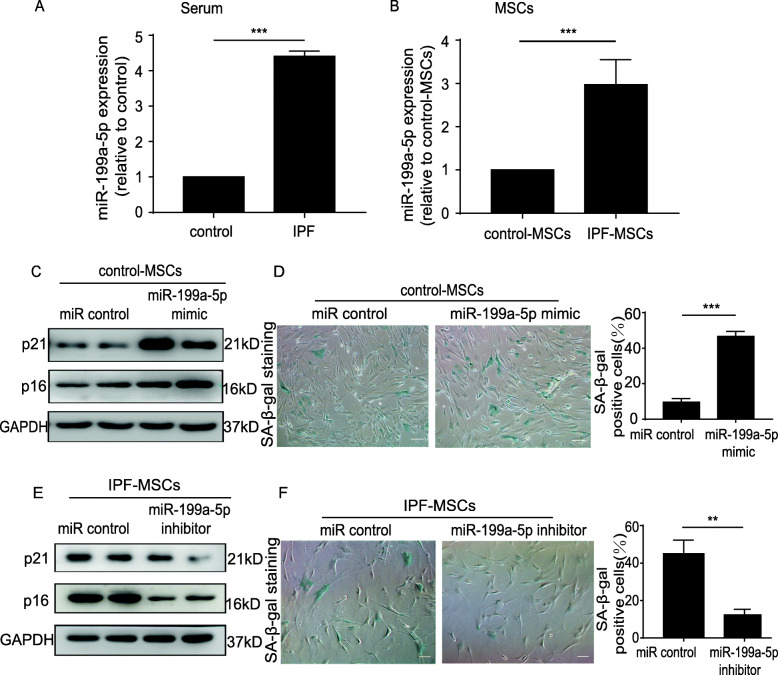
miR-199a-5p induced cellular senescence of MSCs. **a** Expression of miR-199a-5p measured in serum from control donors and patients. **b** Expression of miR-199a-5p measured in control-MSCs and IPF-MSCs. **c** Western blotting analysis of p21 and p16 protein expression in control-MSCs treated with miR control or miR-199a- 5p mimic. **d** Representative images and quantitative analysis of SA-β-gal staining in control-MSCs transfected with miR control or miR-199a-5p mimic. Scale bar = 200 μm. **e** Western blotting analysis of p21 and p16 protein expression in IPF-MSCs treated with miR control or miR-199a- 5p inhibitor. **f** Representative images and quantitative analysis of SA-β-gal staining in IPF-MSCs transfected with miR control or miR-199a-5p inhibitor. Scale bar = 200 μm. All data were obtained from at least three independent experiments and each error bar represents the mean ± SEM. **p < 0.01; ***p < 0.001

A large body of evidence has shown that miRNAs are involved in the regulation of cellular senescence [22, 23]. It has been reported that miR-199a-5p level is significantly altered in patients with IPF and animal models of IPF [17]. We focused on determining whether miR-199a-5p is involved in regulating IPF-MSC senescence. We first measured the level of miR-199a-5p in serum from healthy donors and IPF patients by qRT-PCR. The level of miR-199a-5p was significantly upregulated in IPF patients compared with control donors (Fig. 3a). Compared with control-MSCs, the miR-199a-5p level was also greatly enhanced in IPF-MSCs (Fig. 3b), indicating that the expression of miR-199a-5p was associated with IPF-MSC senescence. Next, we transfected miR-199a-5p mimic directly into control-MSCs to verify the role of miR-199a-5p in regulation of MSC senescence. As shown in Fig. S2A, the miR-199a-5p level was robustly increased in miR-199a-5p mimic-treated control-MSCs (Fig. S2A). Compared with miR control treatment, the expression of p21 and p16 (Fig. 3c) and the level of SA-β-gal activity (Fig. 3d) were dramatically increased in miR-199a-5p mimic-treated control-MSCs. Furthermore, miR-199a-5p mimic treatment significantly reduced the proliferation of control-MSCs as evidenced by the reduction of Ki67-positive cells (Fig. S2B). In addition, we treated IPF-MSCs with a miR-199a-5p inhibitor. The results showed that the miR-199a-5p inhibitor led to significant downregulation of the miR-199a-5p level (Fig. S2C), p21 and p16 protein expression (Fig. 3e) and SA-β-gal activity (Fig. 3f) in IPF-MSCs. Furthermore, Ki67 staining showed that miR-199a-5p inhibitor treatment significantly improved the proliferative rate of IPF-MSCs (Fig. S2D). Collectively, these results indicate that miR-199a-5p mediated the cellular senescence of IPF-MSCs.

### miR-199a-5p induces cellular senescence of MSCs by regulating autophagy

**Fig. 4:**
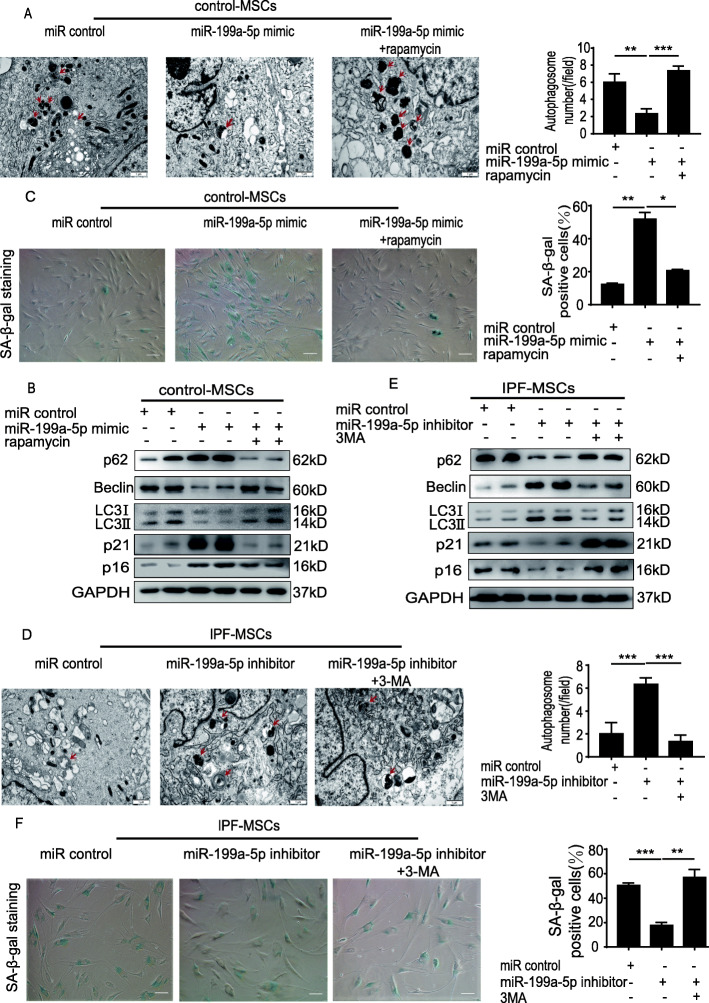
miR-199a-5p induced IPF-MSC senescence by regulating autophagy. **a** Representative images of autophagosomes examined using a TEM and quantitative analysis of autophagosomes in control-MSCs transfected with miR control, miR-199a-5p mimic, or miR-199a-5p mimic + rapamycin. Scale bar = 1 μm. **b** Western blotting analysis of p62, Beclin, LC3II/I, p21, and p16 protein expression in control-MSCs transfected with miR control, miR-199a-5p mimic, or miR-199a-5p mimic + rapamycin. **c** Representative images and quantitative analysis of SA-β-gal staining in control-MSCs transfected with miR control, miR-199a-5p mimic, or miR-199a-5p mimic + rapamycin. Scale bar = 200 μm. **d** Representative images of autophagosomes examined using a TEM and quantitative analysis of autophagosomes in IPF-MSCs transfected with miR control, miR-199a-5p inhibitor, or miR-199a-5p inhibitor +3MA. Scale bar = 1 μm. **e** Western blotting analysis of p62, Beclin, LC3II/I, p21, and p16 protein expression in IPF-MSCs transfected with miR control, miR-199a-5p inhibitor, or miR-199a-5p inhibitor +3MA. **f** Representative images and quantitative analysis of SA-β-gal staining in IPF-MSCs transfected with miR control, miR-199a-5p inhibitor, or miR-199a-5p inhibitor +3MA. Scale bar = 200 μm. All data were obtained from at least three independent experiments and each error bar represents the mean ± SEM. *p < 0.05; **p < 0.01; ***p < 0.001

It has been shown that autophagy mediates cellular senescence of MSCs [24]. We examined whether miR-199a-5p would induce MSC senescence by regulating autophagy. First, we detected the autophagosomes in control-MSCs and IPF-MSCs using TEM. As shown in Fig. S3A, the number of autophagosomes in IPF-MSCs was greatly decreased compared with that in control-MSCs (Fig. S3A). Next, we evaluated the protein expression of some key autophagy-associated proteins including Beclin, LC3II/I, and p62. Western blotting analysis showed that compared with control-MSCs, the expression of Beclin and LC3II/I was greatly decreased in IPF-MSCs, whereas that of p62 was increased (Fig. S3B), suggesting that autophagy was decreased in IPF-MSCs. Subsequently, we treated control-MSCs with miR-199a-5p mimic and found that the number of autophagosomes was much lower than that in miR control-treated control-MSCs (Fig. 4a). In addition, the p62, p21, and p16 expression levels were significantly increased, whereas Beclin and LC3II/I expression was decreased in miR-199a-5p mimic-treated MSCs (Fig. 4b). Moreover, miR-199a-5p mimic treatment enhanced SA-β-gal activity in control-MSCs (Fig. 4c). Compared with miR control, fewer Ki67-positive cells were detected in miR-199a-5p mimic-treated control-MSCs (Fig. S3C). Notably, these effects were partially reversed by rapamycin (an autophagy activator) treatment. Furthermore, compared with miR control-treated IPF-MSCs, miR-199a-5p inhibitor treatment increased the number of autophagosomes (Fig. 4d). Moreover, the expression of Beclin and LC3II/I was significantly upregulated in miR-199a-5p inhibitor-treated IPF-MSCs, while the level of p62, p21, and p16 protein was downregulated (Fig. 4e). As shown in Fig. 4f, treatment with miR-199a-5p inhibitor alleviated the senescence of IPF-MSCs, as evidenced by the result of SA-β-gal staining (Fig. 4f). Compared with the miR control group, more Ki67-positive cells were detected in the miR-199a-5p inhibitor treated group (Fig. S3D). Nonetheless, these effects were partially abrogated by treatment with 3-MA (an autophagy inhibitor) that significantly downregulated the increased autophagosomes and upregulated the decreased cellular senescence of miR-199a-5p inhibitor treated- IPF-MSCs (Fig. 4d–f). These data demonstrate that miR-199a-5p induces cellular senescence of MSCs by regulating autophagy.

### miR-199a-5p regulates autophagy via the Sirt1/AMPK signaling pathway

**Fig. 5:**
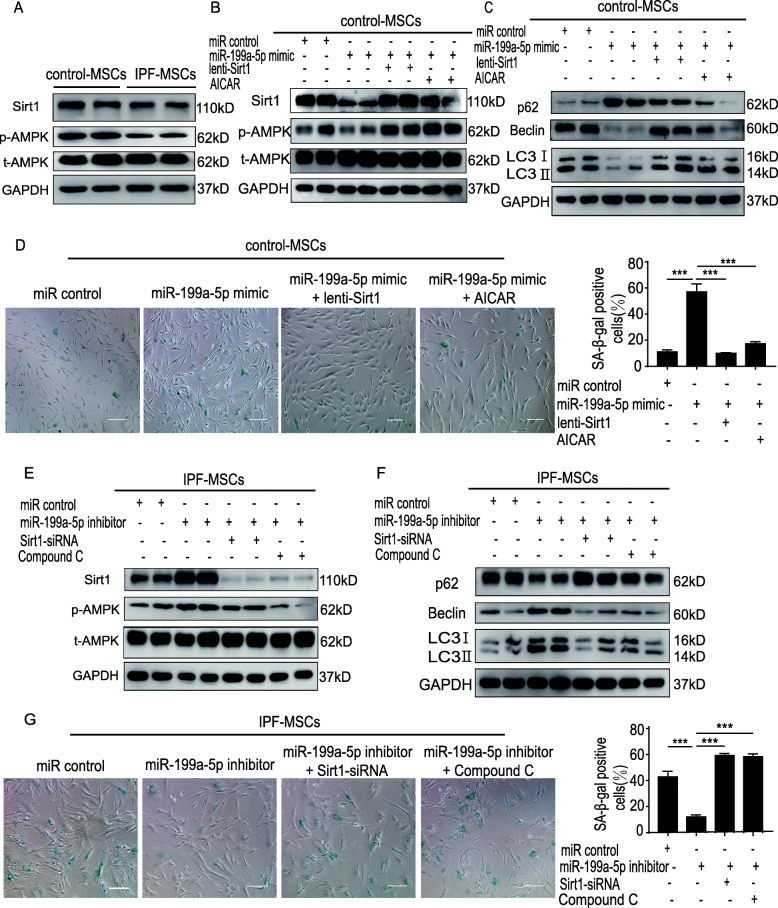
miR-199a-5p regulated autophagy via the Sirt1/AMPK signaling pathway. **a** Western blotting analysis of the expression of Sirt1 and p-AMPK in control-MSCs and IPF-MSCs. **b** Western blotting analysis of the expression of Sirt1 and p-AMPK in control-MSCs treated with miR control, miR-199a-5p mimic, miR-199a-5p mimic + lenti-Sirt1, or miR-199a-5p mimic + AICAR. **c** Western blotting analysis of p62, Beclin, and LC3II/I expression in control-MSCs treated with miR control, miR-199a-5p mimic, miR-199a-5p mimic + lenti-Sirt1, or miR-199a-5p mimic +AICAR. **d** Representative images and quantitative analysis of SA-β-gal staining in control-MSCs treated with miR control, miR-199a-5p mimic, miR-199a-5p mimic + lenti-Sirt1, or miR-199a-5p mimic +AICAR. Scale bar = 200 μm. **e** Western blotting analysis of the expression of Sirt1 and p-AMPK in IPF-MSCs treated with miR control, miR-199a-5p inhibitor, miR-199a-5p inhibitor+Sirt1-siRNA, or miR-199a-5p inhibitor + Compound C. **f** Western blotting analysis of p62, Beclin, and LC3II/I expression level in IPF-MSCs treated with miR control, miR-199a-5p inhibitor, miR-199a-5p inhibitor+Sirt1-siRNA, or miR-199a-5p inhibitor + Compound C. **g** Representative images and quantitative analysis of SA-β-gal staining in IPF-MSCs treated with miR control, miR-199a-5p inhibitor, miR-199a-5p inhibitor + Sirt1-siRNA, or miR-199a-5p inhibitor + compound C. All data were obtained from at least three independent experiments and each error bar represents the mean ± SEM. Scale bar = 200 μm. ***p < 0.001

Since evidence shows that the AMPK signaling pathway regulates autophagy [25, 26], we aimed to determine whether miR-199a-5p mediates autophagy by regulating that pathway. It has also been reported that Sirt1 can affect AMPK activation to mediate autophagy [27, 28]. First, we sought to identify a potential binding sequence for miR-199a-5p within the sequence of Sirt1 using TargetScan (http://www.targetscan.org/). The potential miR-199a-5p binding sequence of Sirt1 3′UTR is shown in Fig. S4A, revealing that Sirt1 is a potential target of miR-199a-5p (Fig. S4A). Next, we tested whether miR-199a-5p overexpression could affect the expression of Sirt1 in control-MSCs. Treatment with miR-199a-5p mimic significantly decreased Sirt1 protein expression (Fig. S4B). Furthermore, the dual-luciferase reporter assays demonstrated that miR-199a-5p mimic transfection significantly reduced the luciferase activity of Sirt1 wild-type (WT) reporter but had no effect on the activity of the Sirt1 mutant reporter (Fig. S4C). These results demonstrated that miR-199a-5p regulates Sirt1 expression. Subsequently, we examined the expression of Sirt1 and p-AMPK in control-MSCs and IPF-MSCs. Compared with control-MSCs, the expression of Sirt1 and p-AMPK was robustly decreased in IPF-MSCs (Fig. 5a). Next, we found that miR-199a-5p mimic treatment downregulated the expression of Sirt1, p-AMPK, Beclin, and LC3II/I but upregulated that of p62 in control-MSCs, suggesting that the Sirt1/AMPK signaling pathway is involved in miR-199a-5p and regulates autophagy (Fig. 5b, c). Nonetheless, these effects were partially reversed by overexpression of Sirt1 or AICAR treatment, AMPK activator in miR-199a-5p mimic-treated control-MSCs. In addition, overexpression of Sirt1 or AICAR treatment attenuated miR-199a-5p mimic-induced senescence of control-MSCs (Fig. 5d). To further verify that miR-199a-5p regulates autophagy via the Sirt1/AMPK signaling pathway, we treated IPF-MSCs with miR-199a-5p inhibitor. Western blotting analysis revealed significantly increased expression of Sirt1, p-AMPK, Beclin, and LC3II/I but decreased expression of p62 in IPF-MSCs (Fig. 5e, f). Notably, these effects were partially abrogated by Sirt1-siRNA or compound C treatment or AMPK inhibitor in miR-199a-5p inhibitor-treated IPF-MSCs (Fig. 5e, f). Moreover, Sirt1-siRNA or compound C treatment reversed the inhibition of cellular senescence in miR-199a-5p inhibitor-treated IPF-MSCs as demonstrated by SA-β-gal staining (Fig. 5g). Collectively, these results suggest that miR-199a-5p regulates autophagy to mediate MSC senescence via the Sirt1/AMPK signaling pathway.

### Transplantation of anti-miR-199a-5p IPF-MSCs ameliorated the symptoms of pulmonary fibrosis

**Fig. 6:**
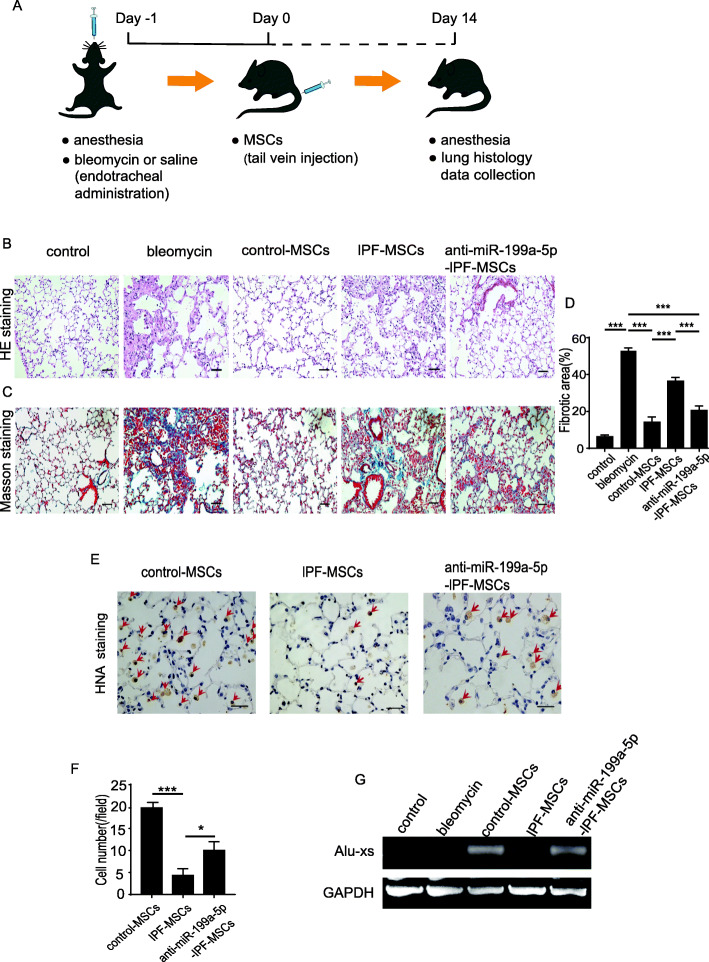
Anti-miR-199a-5p-IPF-MSC transplantation ameliorated the symptoms of pulmonary fibrosis. **a** Schematic chart showing the introduction of bleomycin-induced pulmonary fibrosis and transplantation of control-MSCs, IPF-MSCs, or anti-miR-199a-5p-IPF-MSCs. **b** HE staining of the lung tissue from control mice, bleomycin-treated mice, bleomycin-treated mice that received control-MSCs, IPF-MSCs, or anti-miR-199a-5p-IPF-MSCs. Scale bar = 100 μm. **c** Representative images of Masson’s trichrome staining of the lung tissue from control mice, bleomycin-treated mice, bleomycin-treated mice that received control-MSCs, IPF-MSCs, or anti-miR-199a-5p-IPF-MSCs. Scale bar = 100 μm. **d** Quantitative analysis of fibrosis of the lung tissue from control mice, bleomycin-treated mice, and bleomycin-treated mice that received control-MSCs, IPF-MSCs, or anti-miR-199a-5p-IPF-MSCs. **e** Representative images of HNA staining of the lung tissue from control mice, bleomycin-treated mice, and bleomycin-treated mice that received injections of control-MSCs, IPF-MSCs, or anti-miR-199a-5p-IPF-MSCs. Scale bar = 50 μm. **f** Quantitative analysis of cell survival 14 days post transplantation in bleomycin-treated mice that received control-MSCs, IPF-MSCs, or anti-miR-199a-5p-IPF-MSCs. **g** PCR shows that the expression of Alu-sx was significantly increased in the anti-miR-199a-5p-IPF-MSC group compared with the IPF-MSC group. Data represent the mean ± SEM from groups of six mice. *p < 0.05; ***p < 0.001

To examine whether inhibition of miR-199a-5p in IPF-MSCs could improve the therapeutic effects of IPF-MSCs, we transplanted anti-miR-199a-5p-IPF-MSCs into a mouse model of pulmonary fibrosis. The experimental protocol is outlined in Fig. 6a. As shown in Fig. 6b, HE staining showed that compared with the control group, mice with pulmonary fibrosis displayed serious damage to the lung alveoli structure and a high level of inflammatory cell infiltration. In contrast, the pathological structure of lung tissue was greatly improved in all MSC-treated groups compared with the bleomycin group, with the most significant improvement evident in the control-MSC group. Notably, the structural integrity of lung tissue in the anti-miR-199a-5p-IPF-MSC group was much better than that of the IPF-MSC group (Fig. 6b). Consistent with these findings, a similar result was shown for Masson’s trichrome staining in different MSC-treated groups. The fibrotic area was much higher in the IPF-MSC group than in the control-MSC group but significantly reduced in the anti-miR-199a-5p-IPF-MSC group compared with the IPF-MSC group (Fig. 6c, d). Next, we examined the survival of MSCs in the lung tissue from different groups at 14 days post transplantation. Human nuclear antigen (HNA) immunohistochemical staining showed that although the number of surviving MSCs was highest in lung tissue from the control-MSC group, and MSC survival was much higher in the anti-miR-199a-5p-IPF-MSC group compared with the IPF-MSC group (Fig. 6e, f). To further verify MSC survival in the lung tissue after transplantation, we performed PCR to examine the human repeat sequences Alu-sx in the lung tissue from different groups. As shown in Fig. 6g, Alu-sx was detected in all MSC groups but not in the control or bleomycin group (Fig. 6g). Furthermore, although expression of Alu-sx was highest in the lung tissue from the control-MSC group, it was significantly increased in the anti-miR-199a-5p-IPF-MSC group compared with the IPF-MSC group (Fig. 6g).

### Transplantation of anti-miR-199a-5p-IPF-MSCs attenuated fibrosis formation and inflammation in a mouse model of pulmonary fibrosis

**Fig. 7:**
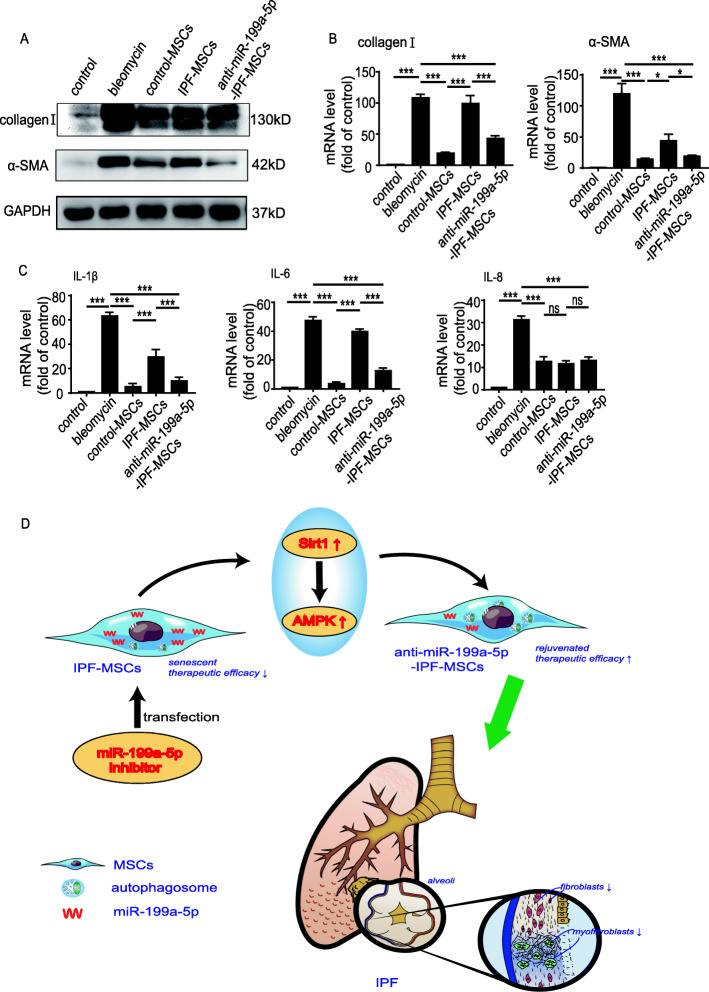
Transplantation of anti-miR-199a-5p-IPF-MSCs attenuated fibrosis formation and inflammation in a mouse model of pulmonary fibrosis. **a** Western blotting analysis of collagen I and α-SMA expression in lung tissue from control group mice, bleomycin group mice, and bleomycin group mice that received control-MSCs, IPF-MSCs, or anti-miR-199a-5p-IPF-MSCs, respectively. **b** Measurement of the mRNA level of collagen I and α-SMA expression in the lung tissue from control group mice, bleomycin group mice, control-MSC group mice, IPF-MSC group mice, or anti-miR-199a-5p-IPF-MSCs group mice. **c** Measurement of mRNA level of proinflammatory genes in the lung tissue from control group mice, bleomycin group mice, control-MSC group mice, IPF-MSC group mice, or anti-miR-199a-5p-IPF-MSC group mice. **d** IPF-MSCs demonstrate increased cellular senescence and decreased therapeutic function. After inhibition by miR-199a-5p, the vitality of IPF-MSCs was restored by regulating autophagy level by activating Sirt1/AMPK signaling pathway. Anti-miR-199a-5p-IPF-MSCs had greater antifibrotic effects in a mouse model of pulmonary fibrosis induced by bleomycin than IPF-MSCs. Data represent the mean ± SEM from groups of six mice. *p < 0.05; **p < 0.01; ***p < 0.001

The protein expression of collagen I and α-SMA was significantly increased in the bleomycin group compared with the control group, but dramatically reduced in the MSC-treated group (Fig. 7a). Notably, the protein expression of collagen I and α-SMA was much lower in the anti-miR-199a-5p-IPF-MSC group than in the IPF-MSC group (Fig. 7a). Similar results were shown for the mRNA level of collagen I and α-SMA. As shown in Fig. 7b, compared with the bleomycin group, the mRNA level of collagen I and α-SMA was greatly reduced in the MSC-treated groups (Fig. 7b). Notably, the mRNA level of collagen I and α-SMA was much lower in the anti-miR-199a-5p-IPF-MSC group than in the IPF-MSC group (Fig. 7b). In addition, the level of IPF associated inflammatory cytokines showed the same trend. Compared with the bleomycin group, the mRNA level of IL-1β, IL-6, and IL-8 was decreased in MSC-treated groups (Fig. 7c). Furthermore, these mRNA levels of IL-1β and IL-6 were lower in the anti-miR-199a-5p-IPF-MSC group than in the IPF-MSC group (Fig. 7c). Nonetheless, there was no significant difference in the mRNA level of IL-8 between the anti-miR-199a-5p-IPF-MSC group and the IPF-MSC group (Fig. 7c). These results indicate that anti-miR-199a-5p-IPF-MSCs were superior to IPF-MSCs in attenuation of fibrosis formation and inflammation in the mouse model of pulmonary fibrosis.

## Discussion

There were several major findings in the current study (Fig. 7d). First, miR-199a-5p-mediated MSC senescence in patients with IPF. Second, miR-199a-5p induced IPF-MSC senescence by regulating autophagy. Third, miR-199a-5p regulated autophagy by targeting the Sirt1/AMPK signaling pathway. Finally, inhibition of miR-199a-5p rejuvenated IPF-MSCs and increased their capacity to prevent lung fibrosis progression induced by bleomycin in mice.

Although MSC-based therapy has shown promising results for treatment of IPF, some drawbacks need to be overcome prior to its clinical application. A major challenge is that MSCs isolated from aged donors or patients are easily senescent, resulting in minimal therapeutic effectiveness [29, 30]. Compared with MSCs derived from age-matched healthy donors, those from patients with abdominal aortic aneurysm exhibit cellular senescence as evidenced by altered multi-differentiation potential, increased SA-β-gal activity and enhanced reactive oxygen species (ROS) production [31]. MSCs derived from diabetic patients also display altered multi-differentiation potential, reduced proliferative capacity and decreased migration, and a senescent phenotype [32]. Since IPF is an age-related disease, MSCs from IPF patients may be also senescent. Indeed, consistent with a previous report [13], in our study, IPF-MSCs demonstrated increased cellular senescence as manifested by decreased proliferation, and increased SA-β-gal activity and expression of p21 and p16. Furthermore, compared with control-MSCs, transplantation of IPF-MSCs showed a decreased therapeutic efficacy for pulmonary fibrosis induced by bleomycin in mice. Nonetheless, the precise mechanisms that underlie IPF-MSC senescence remain unclear. More recently, several strategies including pharmacologic approaches, genetic modification, and cytokine supplementation have been used to rejuvenate senescent MSCs and enhance their beneficial effects [33]. A better understanding of the molecular mechanisms that mediate IPF-MSC senescence will be of great importance when exploring novel strategies for rejuvenation.

Although the underlying mechanisms remain unclear, miRNAs have been reported to play critical roles in regulating MSC senescence. MiR-27b has been shown to contribute to metabolic syndrome-induced adipose tissue-derived MSCs in a porcine model via regulation of the p16/MAPK signal pathway and inhibition of miR-27b downregulated p16 expression and increased MSC migration [15]. MiR-1292 accelerated senescence and restrained osteogenesis of adipose tissue-derived MSCs via the Wnt/β-catenin signaling pathway by targeting FZD4 [34]. The downregulation of miR-1292 reduced senescence and improved osteogenic differentiation. These results reveal that miRNAs mediate MSC senescence. The regulation of some of the key miRNAs is a novel interventional strategy for rejuvenating senescent MSCs. In the current study, the expression of miR-199a-5p was significantly enhanced in the serum of IPF patients and IPF-MSCs. Subsequently, we used loss and gain of function assay and demonstrated that overexpression of miR-199a-5p induced MSC senescence while inhibition rejuvenated IPF-MSCs. Despite this, the precise role of miR-199a-5p in regulating IPF-MSC senescence has not been studied.

Recently, autophagy has been intensively investigated as a major mechanism for MSC senescence. Compared with young-MSCs, the autophagic activity is significantly diminished in aged-MSCs [18]. Moreover, Capasso et al. found that the autophagy was greatly reduced in senescent MSCs induced by oxidative stress, doxorubicin treatment, X-ray irradiation, and replicative exhaustion [35]. In contrast, several studies have shown that activation of autophagy contributes to MSC senescence [36, 37]. High glucose induced MSC senescence by upregulating ROS generation and autophagic activity [38]. These contradictory results suggest that autophagy mediates MSC senescence in a text-dependent manner. In our study, we observed that autophagic activity was decreased in IPF-MSCs compared with control-MSCs. Moreover, overexpression of miR-199a-5p in control-MSCs significantly downregulated autophagic activity, leading to MSC senescence. These effects were partially abrogated by rapamycin. These findings show that miR-199a-5p induces IPF-MSC senescence via regulation of autophagic activity, even though the exact mechanism has not been fully understood.

Previous studies have shown that decreased expression of Sirt1 is closely associated with age-related disease via regulation of autophagy [39, 40]. Sirt1 reverses the cellular senescence of adipose-derived stem cells, induced by oxidative stress, by enhancing autophagy [41]. In this study, we also observed that the expression of Sirt1 was greatly reduced in IPF-MSCs. Further bioinformatics analysis showed that Sirt1 is a potential target of miR-199a-5p, indicating that miR-199a-5p-induced MSC senescence in IPF patients may be due to downregulation of Sirt1. Senescence is usually accompanied by suppression of AMPK and upregulation of AMPK ameliorates cellular senescence [42]. Sirt1 activation of autophagy is linked to the AMPK pathway. The actions of Sirt1 and AMPK are symbiotic; the effect of AMPK can promote Sirt1 activation and Sirt1 can augment the activity of AMPK [43]. We found that overexpression of miR-199a-5p suppressed autophagy in MSCs by downregulating Sirt1/AMPK, leading to cellular senescence, and these effects were partially reversed by overexpression of Sirt1 or AMPK activator. Furthermore, inhibition of miR-199a-5p could rejuvenate IPF-MSCs, and they exhibited an increased therapeutic effect in pulmonary fibrosis induced by bleomycin in mice. In the current study, we examined the therapeutic effect of MSCs on bleomycin-induced pulmonary fibrosis in male mice. It has been established that IPF appears to be more common in men but presents differently in women. Therefore, the therapeutic effect of MSCs on bleomycin-induced pulmonary fibrosis in female mice requires further investigation.

There were several limitations in this study that should be acknowledged. First, despite our novel finding that miR-199a-5p is involved in the regulation of IPF-MSC senescence, it remains unclear whether other altered miRNAs in IPF patients mediate IPF-MSC senescence. Second, in addition to Sirt1/AMPK signaling pathways, whether miR-155-5p regulates other targets to mediate IPF-MSC senescence warrants further investigation. Third, although transplantation of anti-miR-199a-5p-IPF-MSCs was superior to IPF-MSCs in attenuation of pulmonary fibrosis induced by bleomycin in mice, the underlying mechanisms remain elusive. Fourth, the effect of miR-199a-5p on other cell types in IPF such as endothelial cells, epithelial cells, and fibroblasts requires further investigation. Finally, the long-term impact of MSCs on fibrosis formation in a mouse model of pulmonary fibrosis was not determined in the current study.

## Conclusions

These results reveal that miR-199a-5p inhibits autophagy via regulation of Sirt1/AMPK signal pathways, leading to MSC senescence in patients with IPF. The downregulation of miR-199a-5p could rejuvenate IPF-MSC senescence and improve the therapeutic potency of MSC therapy for pulmonary fibrosis. Our study provides a novel candidate target to enhance the therapeutic efficacy of MSC-based therapy for pulmonary fibrosis-related disease.

## Supplementary Information

**Additional file 1: Figure S1.** The plasmid Structure of Sirt1 and anti-miR-199a-5p which contain GFP reporter gene. (A) The plasmid Structure of Sirt1 which contains GFP reporter gene. (B) The plasmid Structure of anti-miR-199a-5p which contains GFP reporter gene. **Figure S2.** miR-199a-5p regulated the proliferation of MSCs. The level of miR-199a-5p in control-MSCs treated with miR control or miR-199a- 5p mimic was measured. (B) Immunostaining of the proliferation marker Ki67 and quantitative analysis of Ki67 positive cells in control-MSCs treated with miR control or miR-199a-5p mimic. Scale bar=100μm. (C) The level of miR-199a-5p in IPF-MSCs treated with miR control or miR-199a- 5p inhibitor was measured. (D) Immunostaining of the proliferation marker Ki67 and quantitative analysis of Ki67 positive cells in IPF-MSCs treated with miR control or miR-199a-5p inhibitor. All data were obtained from at least three independent experiments and each error bar represents the mean ± SEM. Scale bar=100μm. **p <0 .01; ***p <0 .001. **Figure S3.** miR-199a-5p regulated the proliferation of MSCs by regulating autophagy. Representative images of autophagosomes examined using a TEM and quantitative analysis of autophagosomes in control-MSCs and IPF-MSCs. Scale bar=1μm. (B) Western blotting analysis of p62, Beclin, and LC3II/I protein expression in control-MSCs and IPF-MSCs. (C) Immunostaining of the proliferation marker Ki67 and quantitative analysis of Ki67 positive cells in control-MSCs transfected with miR control, miR-199a-5p mimic, or miR-199a-5p mimic + rapamycin. Scale bar=100μm. (D)Immunostaining of the proliferation marker Ki67 and quantitative analysis of Ki67 positive cells in lPF-MSCs transfected with miR control, miR-199a-5p inhibitor, or miR-199a-5p inhibitor + 3MA. All data were obtained from at least three independent experiments and each error bar represents the mean ± SEM. Scale bar=100μm. *n* = 3. **p <0 .01; ***p <0 .001. **Figure S4.** The *3*′*UTR of Sirt1 contains one binding site for miR-199a-5p.* (A) Predicted *binding site for miR*-*199a-5p* within the *3*′*UTR* sequence *of* Sirt1. (B) Western blotting analysis of the expression level of Sirt1 in control-MSCs treated with miR control, miR-199a-5p mimic. (C) The luciferase reporter vector containing WT Sirt1 3′UTR or mutant 3′UTR was cotransfected with miR-199a-5p mimic or miRNA control into HEK293 cells. All data were obtained from at least three independent experiments and each error bar represents the mean ± SEM. ***p <0.001; ns: not significant.

## Data Availability

All data has been included in the paper and supplement. The datasets used in this study are available from the corresponding author on reasonable request.

## Abbreviations

BCA: Bicinchoninic acid
DLCO: Carbon monoxide diffusing capacity
FVC: Forced vital capacity
HNA: Human nuclear antigen
IPF: Idiopathic pulmonary fibrosis
MSC: Mesenchymal stem cell
ROS: Reactive oxygen species
SA-β-gal: Senescence-associated β-galactosidase
Sirt1: Sirtuin 1
TEM: Transmission electron microscope
UTR: Untranslated region
WT: Wild-type
