# Supplementary material for: Inhibition of miR-199a-5p rejuvenates aged mesenchymal stem cells derived from patients with idiopathic pulmonary fibrosis and improves their therapeutic efficacy in experimental pulmonary fibrosis
Source: Stem Cell Res Ther. 2021 Feb 25;12:147. doi: 10.1186/s13287-021-02215-x (PMC7905557; doi:10.1186/s13287-021-02215-x)

**Supplement Figure 1: The plasmid Structure of Sirt1 and anti-miR-199a-5p which contain GFP reporter gene.**

(A) The plasmid Structure of Sirt1 which contains GFP reporter gene. (B) The plasmid Structure of anti-miR-199a-5p which contains GFP reporter gene.


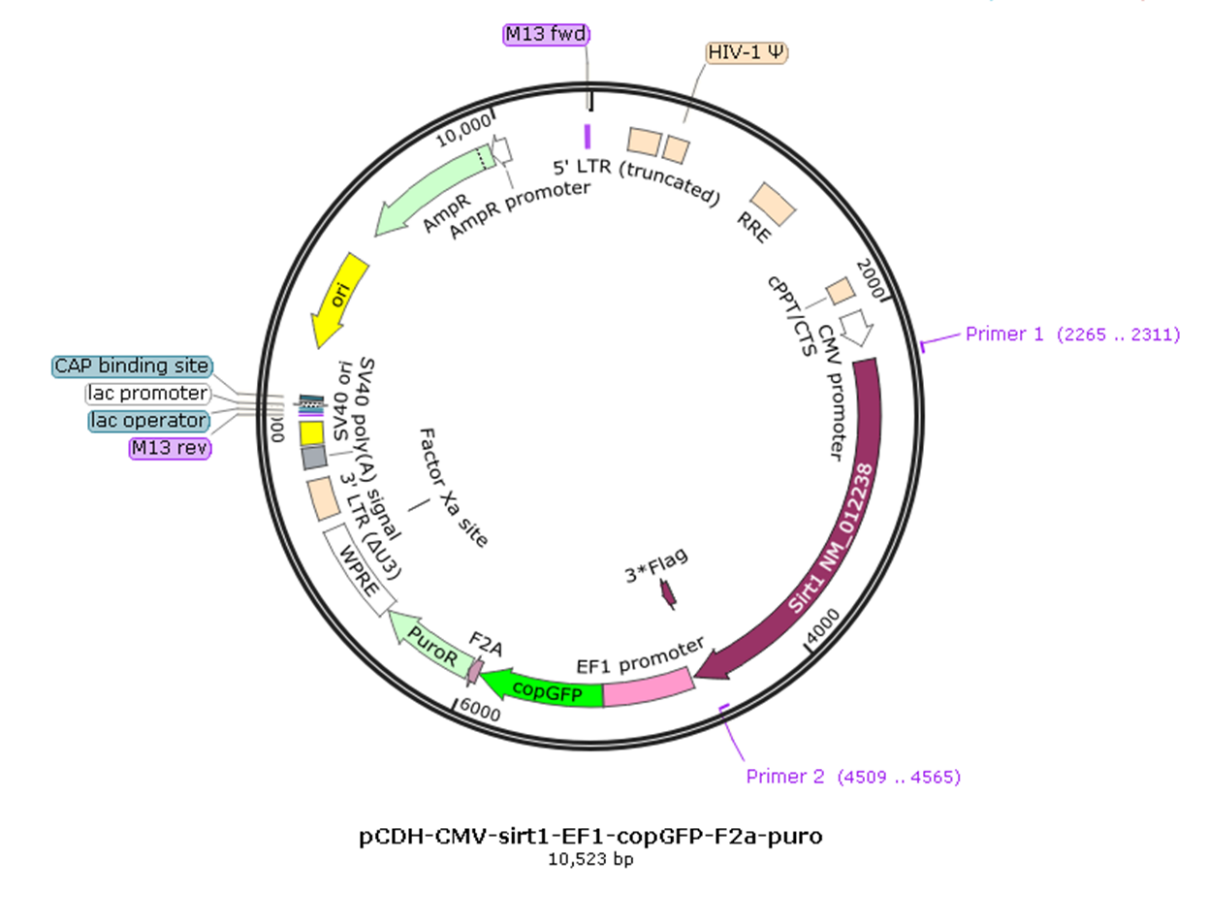
A

B

**
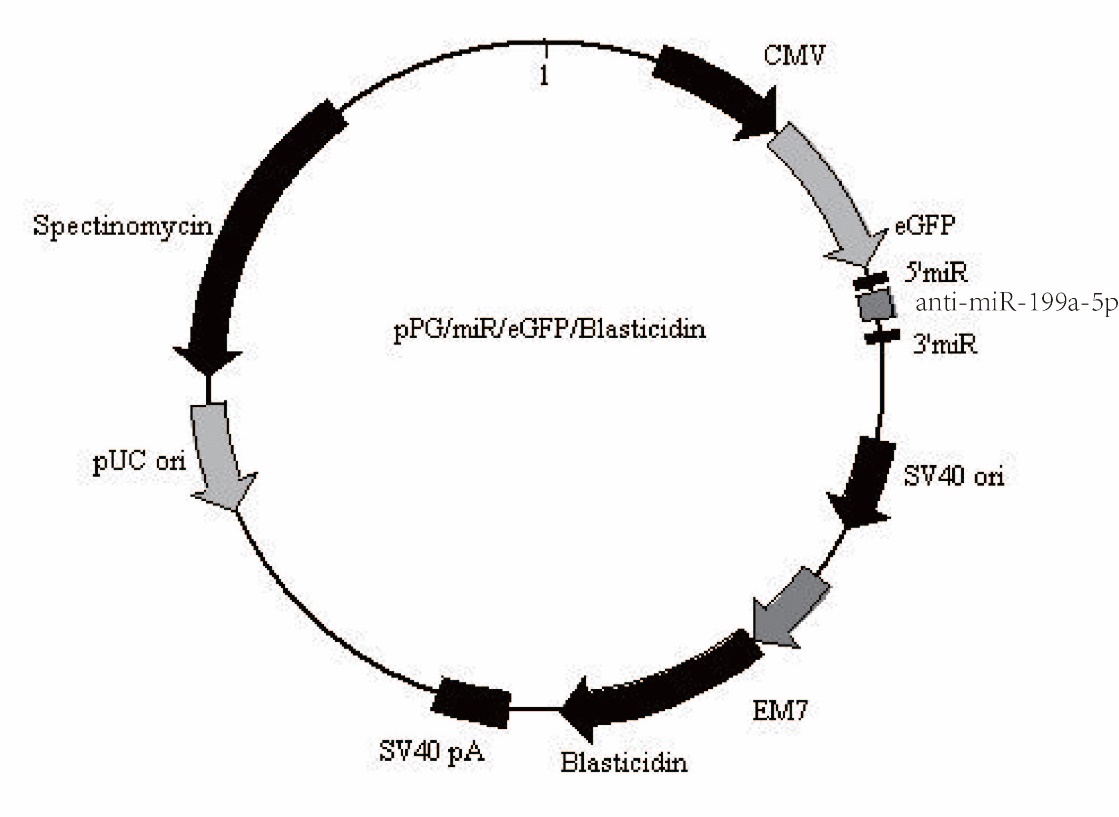
Supplement Figure 2: miR-199a-5p regulated the proliferation of MSCs**.

1. The level of miR-199a-5p in control-MSCs treated with miR control or miR-199a- 5p mimic was measured. (B) Immunostaining of the proliferation marker Ki67 and quantitative analysis of Ki67 positive cells in control-MSCs treated with miR control or miR-199a-5p mimic. Scale bar=100μm. (C) The level of miR-199a-5p in IPF-MSCs treated with miR control or miR-199a- 5p inhibitor was measured. (D) Immunostaining of the proliferation marker Ki67 and quantitative analysis of Ki67 positive cells in IPF-MSCs treated with miR control or miR-199a-5p inhibitor. All data were obtained from at least three independent experiments and each error bar represents the mean ± SEM. Scale bar=100μm. ***p* <0 .01; ****p* <0 .001.


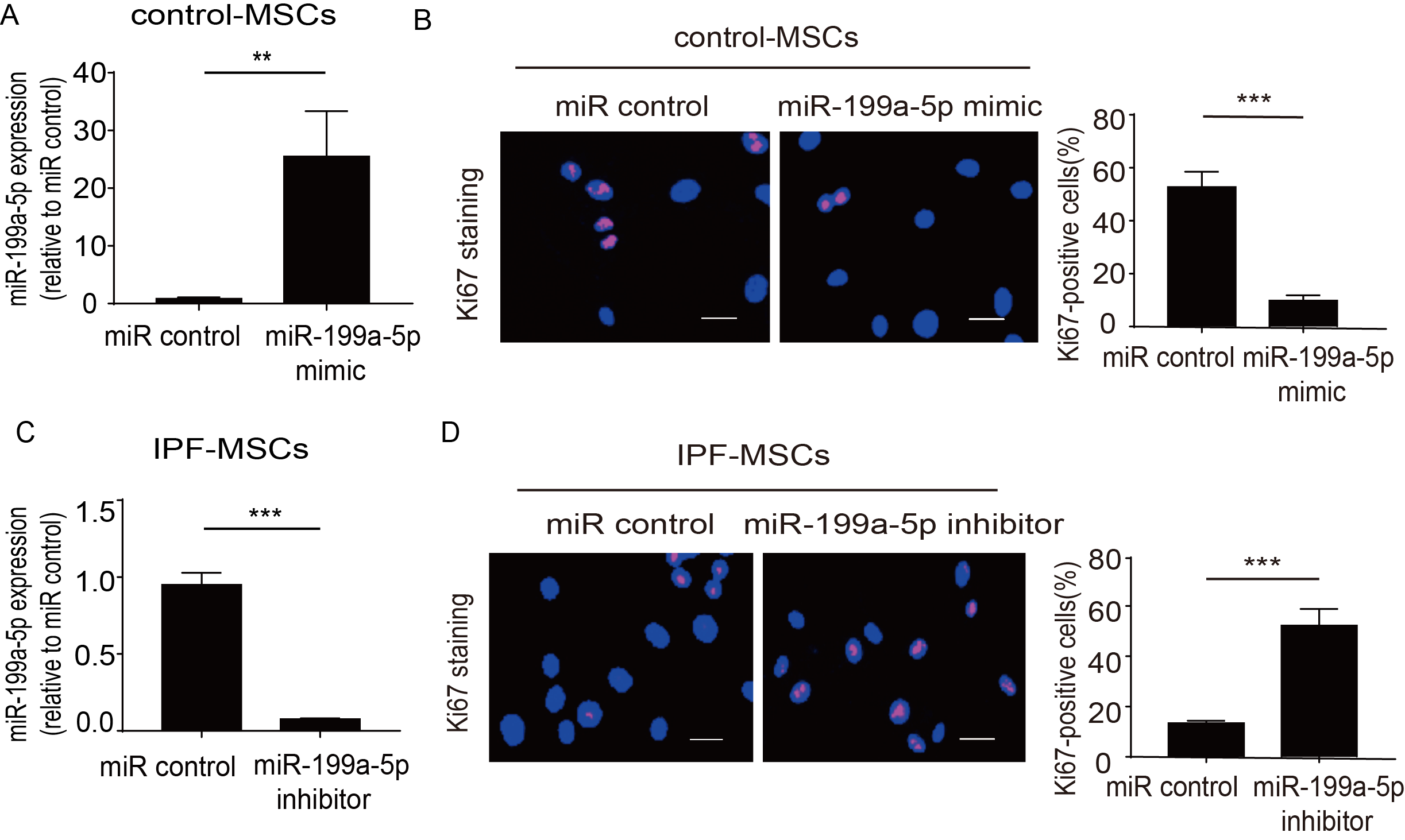


**Supplement Figure 3: miR-199a-5p regulated the proliferation of MSCs by regulating autophagy.**

1. Representative images of autophagosomes examined using a TEM and quantitative analysis of autophagosomes in control-MSCs and IPF-MSCs. Scale bar=1μm. (B) Western blotting analysis of p62, Beclin, and LC3II/I protein expression in control-MSCs and IPF-MSCs. (C) Immunostaining of the proliferation marker Ki67 and quantitative analysis of Ki67 positive cells in control-MSCs transfected with miR control, miR-199a-5p mimic, or miR-199a-5p mimic + rapamycin. Scale bar=100μm. (D)Immunostaining of the proliferation marker Ki67 and quantitative analysis of Ki67 positive cells in lPF-MSCs transfected with miR control, miR-199a-5p inhibitor, or miR-199a-5p inhibitor + 3MA. All data were obtained from at least three independent experiments and each error bar represents the mean ± SEM. Scale bar=100μm. *n* = 3. ***p* <0 .01; ****p* <0 .001.

**
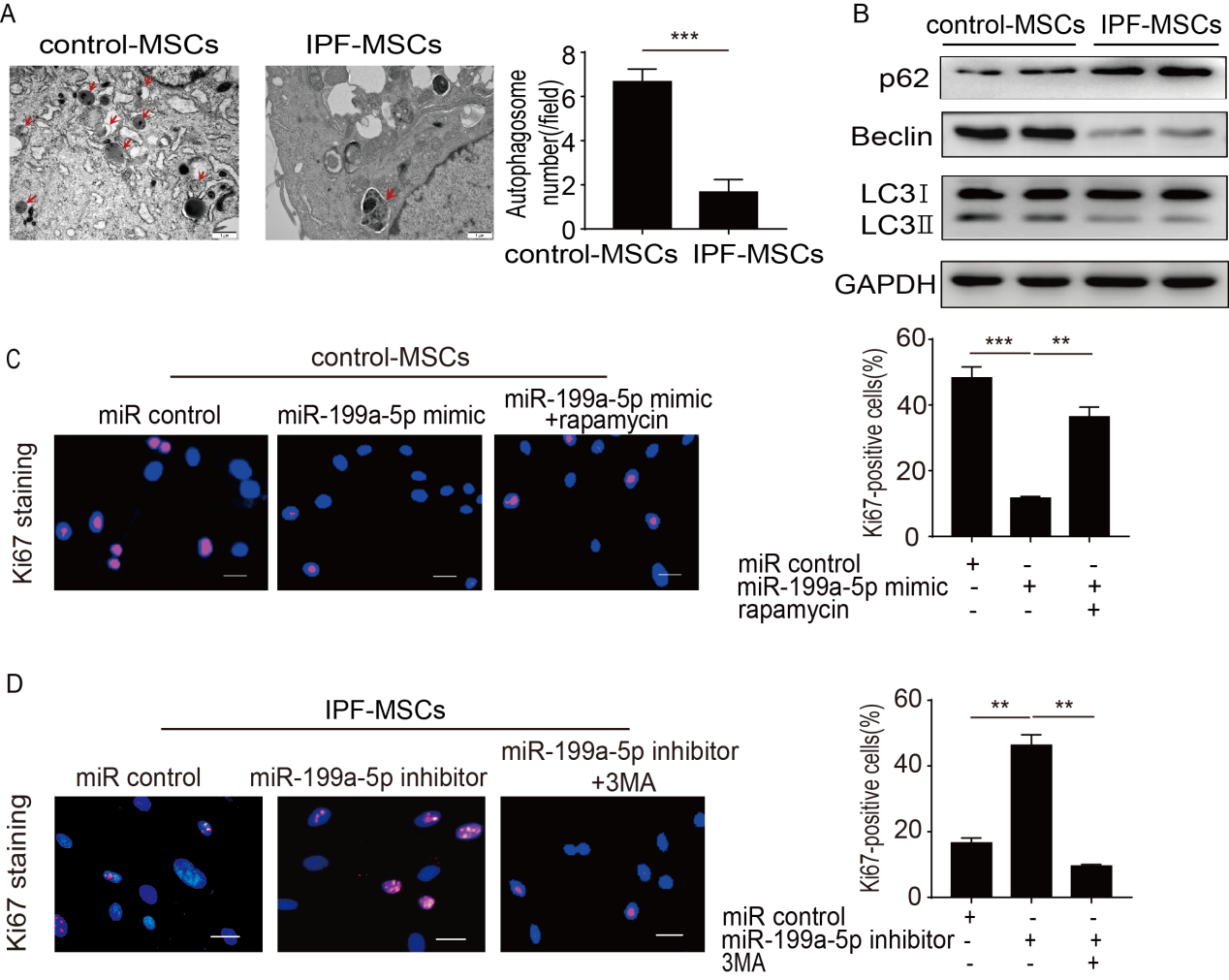
**

**Supplement Figure 4: The 3′UTR of Sirt1 contains one binding site for miR-199a-5p.**

(A) Predicted binding site for miR-199a-5p within the 3′UTR sequence of Sirt1. (B) Western blotting analysis of the expression level of Sirt1 in control-MSCs treated with miR control, miR-199a-5p mimic. (C) The luciferase reporter vector containing WT Sirt1 3′UTR or mutant 3′UTR was cotransfected with miR-199a-5p mimic or miRNA control into HEK293 cells. All data were obtained from at least three independent experiments and each error bar represents the mean ± SEM. ****p <0.001;* ns: not significant.


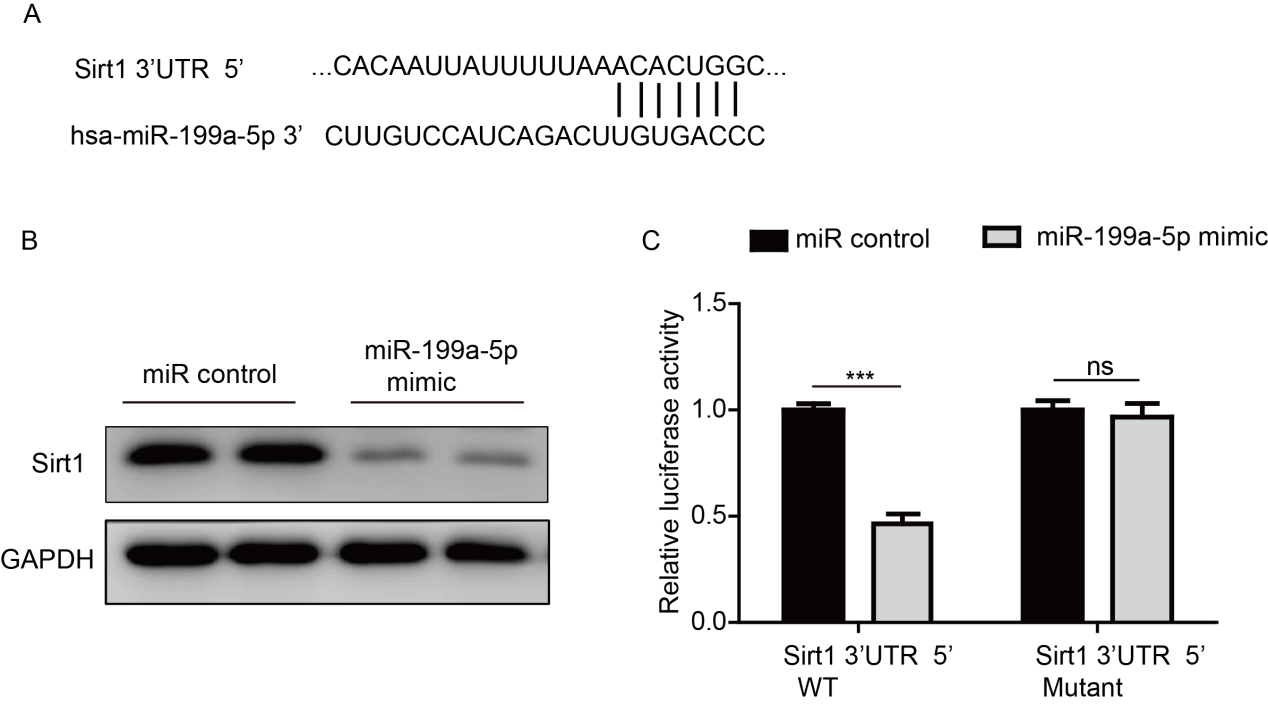

Supplement: Supplementary file 1 — Additional file 1: Figure S1. The plasmid Structure of Sirt1 and anti-miR-199a-5p which contain GFP reporter gene. (A) The plasmid Structure of Sirt1 which contains GFP reporter gene. (B) The plasmid Structure of anti-miR-199a-5p which contains GFP reporter gene. Figure S2. miR-199a-5p regulated the proliferation of MSCs. The level of miR-199a-5p in control-MSCs treated with miR control or miR-199a- 5p mimic was measured. (B) Immunostaining of the proliferation marker Ki67 and quantitative analysis of Ki67 positive cells in control-MSCs treated with miR control or miR-199a-5p mimic. Scale bar=100μm. (C) The level of miR-199a-5p in IPF-MSCs treated with miR control or miR-199a- 5p inhibitor was measured. (D) Immunostaining of the proliferation marker Ki67 and quantitative analysis of Ki67 positive cells in IPF-MSCs treated with miR control or miR-199a-5p inhibitor. All data were obtained from at least three independent experiments and each error bar represents the mean ± SEM. Scale bar=100μm. **p <0 .01; ***p <0 .001. Figure S3. miR-199a-5p regulated the proliferation of MSCs by regulating autophagy. Representative images of autophagosomes examined using a TEM and quantitative analysis of autophagosomes in control-MSCs and IPF-MSCs. Scale bar=1μm. (B) Western blotting analysis of p62, Beclin, and LC3II/I protein expression in control-MSCs and IPF-MSCs. (C) Immunostaining of the proliferation marker Ki67 and quantitative analysis of Ki67 positive cells in control-MSCs transfected with miR control, miR-199a-5p mimic, or miR-199a-5p mimic + rapamycin. Scale bar=100μm. (D)Immunostaining of the proliferation marker Ki67 and quantitative analysis of Ki67 positive cells in lPF-MSCs transfected with miR control, miR-199a-5p inhibitor, or miR-199a-5p inhibitor + 3MA. All data were obtained from at least three independent experiments and each error bar represents the mean ± SEM. Scale bar=100μm. n = 3. **p <0 .01; ***p <0 .001. Figure S4. The 3′UTR of Sirt1 contains one bindin [file 13287_2021_2215_MOESM1_ESM.docx]
